# Research on the Morphology of the Working Surfaces of Contacts Used in Starters in the Agro-Industrial Sector

**DOI:** 10.3390/ma17010145

**Published:** 2023-12-27

**Authors:** Volodymyr Korobskyi, Kamil Witaszek, Volodymyr Reshetiuk, Krzysztof Pilarski

**Affiliations:** 1 Department of Electrical Engineering, Electromechanics and Electrotechnology, Education and Research Institute of Energetics, Automatics and Energy Saving, National University of Life and Environmental Sciences of Ukraine Kyiv, Heroyiv Oborony Str., 15, 03-041 Kyiv, Ukraine; kor.vladlen.2002@gmail.com; 2 Department of Biosystems Engineering, Faculty of Environmental and Mechanical Engineering, Poznań University of Life Sciences, Wojska Polskiego 50, 60-627 Poznań, Poland; krzysztof.pilarski@up.poznan.pl; 3 Department of Fundamentals of Engineering and Power Engineering, Institute of Mechanical Engineering, Warsaw University of Life Sciences, Nowoursynowska 166, 02-787 Warsaw, Poland; volodymyr_reshetiuk@sggw.edu.pl

**Keywords:** wear resistance, arc erosion, electric device, contact system, working surface of a contact, silver, copper, environmentally safe composite material, metal–ceramic contact, electron microscope, morphology

## Abstract

The operational suitability of electromagnetic starters equipped with experimental contacts has been substantiated within their use in electrical installations of the agro-industrial sector, which may be affected by the environments containing aggressive components. Tests on commutation wear resistance and investigations on arc erosion of the series-produced contact parts of such starters as PML-1100O4, PML-2100O4 (versions A and B; contact material—CpH-90, CpM-0,2 + M1, KMK-A10m, respectively) and PML-1100O4 starter with the experimental copper-based contact parts (Cu + Nb + Zr + Y_2_O_3_; Cu + Mo + MoO_3_ + C + Ni; Cu + Cr + TiB_2_ + Nb + C + Zr) have been conducted. The influence of energy parameters of a commutated circuit on the value of electro-erosion wear, the morphology of the working surfaces of contacts and contact resistance have been determined. Investigation results have been obtained by conducting a set of tests on electromagnetic starters at the experimental plant that simulates the operating conditions of the AC-3 application category. The impact of the electric arc of alternative current on the arc erosion of silver-based and copper-based contact materials have been determined by using a scanning electron microscope Cambridge Stereoscan S4-10 equipped with an attachment for X-ray spectroscopic analysis, Link System-290 and an X-ray microanalyzer Camebax SX-50 (CAMECA, Gennevilliers, France). A metallographic analysis of the contact surfaces has been conducted, which contributed to the determination of the patterns of erosive destruction of bridging contacts based on Ag and Cu. Evolution of the eroded morphology of contacts and the surface components of electrical contacts under the influence of an arc have been characterized. In addition, contact mass loss and the dependence of contact resistance have been studied. When manufacturing the experimental contacts, it is possible to abandon the use of silver, which is significantly cost saving, and not to use dangerous contact additives that are hazardous to the environment and people’s health.

## 1. Introduction

The operational capacity of an electric contact is mostly determined by its component parts, its structure and the properties of its surface layers that are formed as a result of erosion and transferring contact materials in an electric arc [1,2,3]. The profound study of the physical processes which take place on the working surfaces of contacts during their operation creates preconditions for further struggling with electric erosion, which is one of the primary causes of electric contact damage.

Electric contact materials based on Ag are widely used as contacts in commuting equipment due to their low electrical resistivity and high thermal conductivity. For electromagnetic starters with currents of up to 10 A, the producers mostly use material such as CpH-90 (Ag—90wt%, Ni—10wt%) or CpM-0,2 + M1 (Ag—99.8wt%, Cu—0.2wt%). To produce more powerful starters with the current of 25 A and above, the most popular electrical contact material applied is the one based on silver—KMK-A10m (Ag—85wt%, CdO—15wt%). However, this material has been proven to be extremely harmful to people’s health and the environment [4,5,6]. Therefore, the research and the development of environmentally friendly alternative contact materials that do not contain cadmium is relevant and is gaining sustained attention in the academic field and the industrial sector. Over the past few decades, attempts have been made to substitute CdO with other metal oxides. Most of such investigations have been conducted in the laboratories of countries such as Germany, China, Japan, the USA and Ukraine, showing that the efficient substitute for cadmium oxide can be tin oxide SnO_2_ (according to the impact on the human body, cadmium oxide is classified as a first class hazardous substance, while tin oxide is a substance of hazard class 3) as well as ZnO, CuO and PbO [7,8]. Some scientists recommend to introduce minor additives of zirconium diboride ZrB_2_ and indium oxide In_2_O_3_ into such compositions [9,10]. However, it is known that the disadvantage of the material based on Ag–SnO_2_ is its high contact resistance, which may cause overheating under prolonged loads [11].

The development of contact materials based on copper with additives for switching devices used in agro-industrial electrical installations, namely in livestock facilities, has become of particular relevance. In such applications, the requirement for high contact wear resistance is not as critical. Here, there is a small number of actuations a day that is required, but there is an increased demand for the formation of various films on the surface of a contact that would not impair conductivity, that is, the reliability of contacting. This is caused by the possible presence of aggressive harmful impurities in the environment of agricultural premises.

The options of applying copper-based electrical contact material in the electrical devices for traction stock are covered in the paper [12] for the system of Cu-C-ZrO_2_-TiO_2_-TiB_2_. The influence of technological modes on the production of copper–carbon nanotube composites or copper-based composite contact materials has been described in the papers [13,14], respectively.

One of the options to contribute to the environmental safety, silver savings as well as to meet the requirements of the reliable performance of switching devices in these specific branches is the substitution of silver-based contacts, which contain hazardous impurities, with the contacts made of copper with various additives [15,16].

Due to their low cost, rather high mechanical strength and high values of specific conductivity and thermal conductivity, contacts made of copper, their alloys and composites are widely applied in various kinds of heavily loaded contactors and switches that operate under significant mechanical forces with abrasion and at voltages *U* > 100 B. The hardness and toughness of copper are higher than those of silver. The main drawback of copper is its susceptibility to atmospheric corrosion with the formation of oxide and sulfide films with high resistance, which may disrupt conductivity. Copper is oxidized quickly during heating, but the strength of oxide film adhesion to metal is low and it is disrupted at contact closure and sliding.

Due to its oxidation, pure copper is not suitable for low-current contacts, although the parameters of an electric arc in copper are slightly higher than those in silver. Mechanical detachment and thermal decomposition of an oxide film causes an increased wear of copper-based contacts at high currents. That is why, in modern devices, copper contacts are replaced with contacts made of composite materials or copper-based alloys. A number of elements and compounds can be used as additives. These may include fusible and refractory, rare-earth and precious metals. The most common metallic materials include Ni, In, Bi, Sn, Zn, Fe, Co and Mo. As for the contact material matrix, the most frequently used compounds include oxides, carbides, borides and intermetallic compounds. The additives such as graphite, boron and nitride that can decrease metal susceptibility to welding are applied. In general, alloy additives can be divided into three groups: metals that are unlimitedly dissolved in copper (Ni, Cd); metals with limited solubility (Nb, Ti, Cr, Zr); and metals that do not interact with copper (Mo) [3].

The influence of the direct current electric arc on the erosion of Ag/Ni contact material depending on the number of switching cycles is described in the paper [17]. The results of these authors’ research show that arc erosion at the anode is higher than the one at the cathode. Furthermore, during the first 10 thousand switching cycles, holes and pores initially appeared around the arc influence spot. The morphology of molten silver was different at the cathode and at the anode due to the effect of gravity and arc erosion.

The paper [18] covers the influence of load characteristics (inductive and resistive nature) on the arc erosion of contacts made from Ag-8wt% Ni. The change in contact mass and the morphology of erosion on the surface are influenced by an electric arc of various rebound heights. Furthermore, a resistive load causes significantly less mass loss in comparison with an inductive load and, at the same time, there is less arc power observed. The paper [19] covers the positive impact of adding 4wt%Ni to the contact material Ag-4wt% SnO_2_. In the case of adding Ni, thin distinctive phases form (which is useful for arc scattering) and contact material spattering and the erosion decrease. The morphology of the contact surface made of the material Ag-12wt% Ni is described in more detail in the paper [20], which states that the arc energy and the time of arc burning at the contacts are exponentially related. Arc erosion plays a significant role in the composition and the surface morphology of electrical contact materials, which is mentioned in the paper [12]. The authors state that the changes in surface morphology are derived from charge carriers that may significantly influence arc parameters such as the arc time, the arc energy, the arc power as well as the voltage curve and the arc current.

There are several literature sources which indicate the impact of such additives as Ni, or ZrO_2_ and TiO_2_ that are added to the base material [11,12], or the technological features of obtaining electrical contact material [13,14,18,20,21], or the electrical arc energy [22] on the results of arc erosion of silver contacts. There are some papers that cover the influence of copper-based contacts on arc erosion, namely the influence of copper and tungsten with the use of the reduced graphene oxide in the matrix [11,12], layered composite condensed materials Cu–W [23] and the influence of inter-phase wettability on the composites of Zn_2_SnO_4_/Cu type [24].

Currently, the methods of electron microscopy are widely applied in material science. One of the reasons for this is that in the course of experimentation, it is possible to observe both the image of an object in real space and the diffraction pattern obtained from this object. A scanning microscope is used mostly for investigating the working surfaces of a contact, the crystal structure and the structure of the defects on the surface of contacts. Here, transmission electron microscopes with short wavelengths of radiation are applied. Due to this, particles smaller than 150 nanometers are displayed. Microstructure is one of the main factors that influence the service properties of electrical contacts. It depends on the technology of production, the properties of the initial materials, the design features of a switching device, the value of electric arc energy and the chemical composition of the surrounding atmosphere [25].

The success of applying high-resolution microscopy in material science is due to the fact that there is a possibility to answer the following questions: How distinct is the boundary between various phases? Which nanoscale phases are present in the sample? In some cases, it becomes possible to determine the atomic mechanism by which nanophases transform into one another; this is important for many industries in the study of a structure and phase composition with the example of various materials.

In a transmission electron microscope, the inelastic scattering of electrons leads to the production of characteristic X-ray radiation, by which the elemental composition of the test sample can be determined. Therefore, modern transmission electron microscopes are equipped with attachments for an X-ray spectral microanalysis, which makes it possible to determine the composition of objects up to the atomic level.

Metallographic analysis of the working surfaces of composite contacts contributes to the identification of the patterns of electro-erosion wear, establishing the causes of this or the type of erosion destruction of contacting surfaces. Erosion wear of rupture contacts can be caused by the presence of bridge erosion, the action of a short arc (mass transfer from anode to cathode) or the appearance of a plasma arc (usually mass transfer from cathode to anode). The distinguishing morphological feature of a plasma arc impact is the significant difference in the size of the cathode (a_K_) and the anode spots (a_a_) that remain on the surface of the contacts. It should be pointed out that a_a_ > a_K_, while a_a_ = a_K_ is typical for a short arc.

However, there is limited information in the literature on element distribution and the mechanism of formation of a molten bath of Ag-based contacts after arc erosion. Information on the distribution of elements and the mechanism of molten bath formation is valuable for improving the welding resistance and wear resistance of contacts.

## 2. Materials and Methods

The researchers analyzed series-produced and the experimental bridging contacts of PML starters of the first (up to 10 A) and the second (up to 25 A) sizes. The main component of series-produced contact materials of electromagnetic starters and relays is silver. The experimental contact components are made from environmentally friendly contact materials based on copper with additives:

(1) A total of 83.5% Cu + 15% Nb + 1.0% Zr + 0.5% Y_2_O_3_ [15].
(2) A total of 81.3% Cu + 10% Cr + 3% TiB_2_ + 3% Nb + 2% C + 0.7% Zr [16].

**Figure 1:**
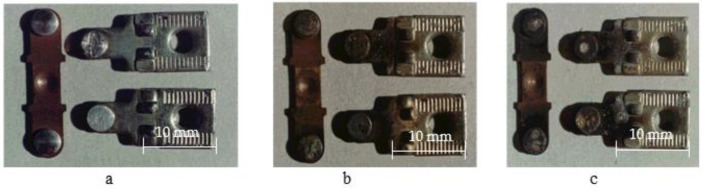
External appearance of serial contacts of PML-1100 O_×_4 B starter after its operation: (**a**) 0 commutation cycles; (**b**) 150 thousand cycles; (**c**) 300 thousand cycles. Category AC-3 (*U* = 380 V; *I* = 10 A). Material: CpM-0,2 + M1. Magnification ×4.

**Figure 2:**
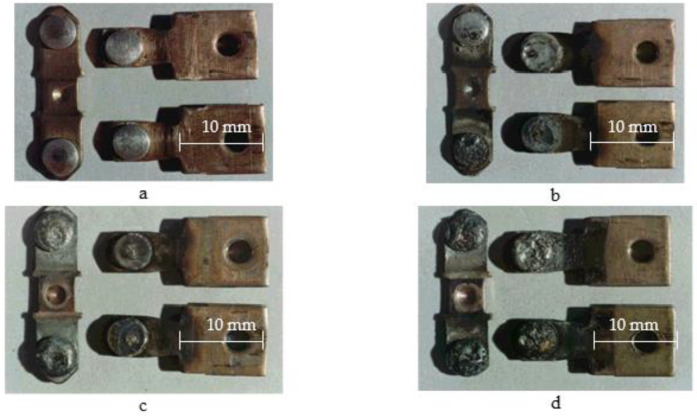
External appearance of serial contacts of PML-2100O_x_4 B starter after its operation: (**a**) 0 commutation cycles; (**b**) 150 thousand cycles; (**c**) 300 thousand cycles; (**d**) 500 thousand cycles. Category AC-3 (*U* = 380 V; *I* = 25 A). Material: KMK-A10m. Magnification ×4.

**Figure 3:**
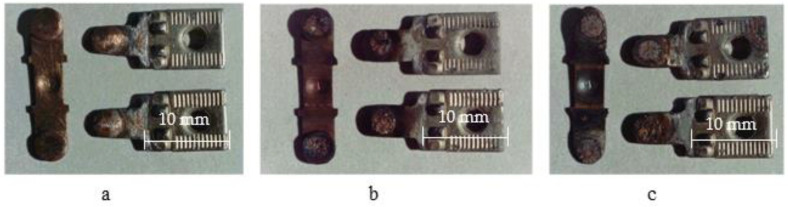
External appearance of experimental contacts of PML-1100 starter after its operation: (**a**) 0 commutation cycles; (**b**) 150 thousand cycles; (**c**) 300 thousand cycles (category AC-3 (*U* = 380 V; *I* = 10 A); magnification ×4; material 81.3% Cu + 10% Cr + 3% TiB_2_ + 3% Nb + 2% C + 0.7% Zr).

The photographs of serial bridging contacts are shown in Figure 1 and Figure 2 while the experimental ones are presented in Figure 3.

Morphological studies of contact surfaces were conducted using the methods found in [25,26,27,28,29], with the use of a scanning electron microscope ‘Cambridge Stereoscan’ S4-10 (Kyiv, Ukraine) equipped with an attachment for X-ray spectroscopic analysis, Link System-290 and an X-ray microprobe analyzer ‘Camebax SX—50’.

The hardness (microhardness) of the phase components of a composite’s sintered materials, referred to as imaginary hardness, was determined using the Vickers method [29] with the PMT-3 device. Measurements were conducted on three samples of each material. A minimum of five measurements were taken on each sample.

Commutation tests were performed for the starters without thermal relays for each contact material in the AC-3 application category [6]. The total number of commutation cycles (switching on and off) for all starters was set at 300,000. The necessary measurements were taken after every 50,000 commutation cycles, and the switching frequency was determined according to the requirements [30]. Current loads were selected for the starters of the first size based on the values of nominal working currents: 4 A, 6.3 A, 10 A. Tests were conducted in the presence of a special corrosive environment, which consisted of a mixture of gases: NH_3_ + H_2_S. The nominal upper concentration values for ammonia and hydrogen sulfide were 30 mg·m^−3^ and 25 mg·m^−3^, respectively.

For conducting experimental tests, the most acceptable method for determining wear is the weight (mass) method. Both moving and stationary contact parts, along with the contact holders, were weighed before and after each series of commutation using analytical scales VLA-200M, which provided a weighing accuracy of no less than 10^−4^ g. Weighing was repeated no less than three times, and the final result was taken as the arithmetic mean. The sample size of starters was set at N = 8 randomly selected starters. Processing of the experimental data to determine the mathematical law of changes in the mass of contact parts as a function of operation was carried out according to the standard methodology as described in paper [2].

Contact resistance of the serial and the experimental contact pairs was determined using a voltmeter–ammeter method, with a total of 300,000 commutation cycles. Measurements of a voltage drop (∆*U*) were taken after every 50,000 cycles with the nominal current of 10 A. A total of 100 measurements of voltage drop values were taken in each commutation series, with the contacts closed for 10 s. All the measurements were conducted using the electrical circuit of the plant. For the starter, the voltage drop was determined for each pole of the main circuit.

The obtained dataset on the voltage drop for the serial contacts CpM-0,2 + M1 and the experimental ones 81.3% Cu + 10% Cr + 3% TiB_2_ + 3% Nb + 2% C + 0.7% Zr was processed by applying the methods of mathematical statistics with the use of ‘Statistika’ program. Contact resistance ∆*R* (mΩ) was calculated using the formula:

(1) $$\Delta R=\frac{\Delta U}{I}$$

where:

∆*U*—Voltage drop at the contact junction, mV;

*I*—Current, A.

Contact bounce and stroke of starters were monitored using standard methods, using an electrical indicator and a linear displacement indicator ICH with a precision of 0.01 mm.

## 3. Results and Discussion

The magnitude of electrical erosion wear was determined based on the change in the mass of contacts, where:

(2) $$m_{2}=m_{1}-k\cdot n$$

where:

*m*_1_—The mass of a contact before the start of commutation tests, g; 

*m*_2_—The mass of a contact after a series of commutation tests, g; 

*k*—Wear intensity coefficient, g/cycle; 

*n*—Number of commutation cycles, cycles.

**Figure 4:**
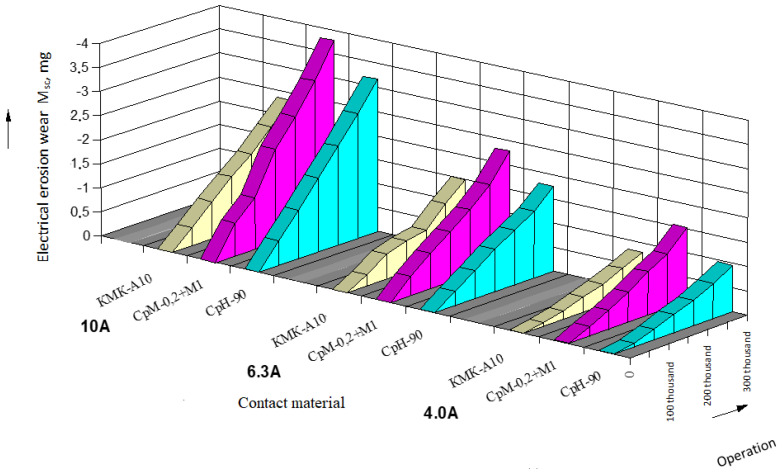
Dependence of electrical erosion wear of serial stationary contact parts of PML starters.

**Figure 5:**
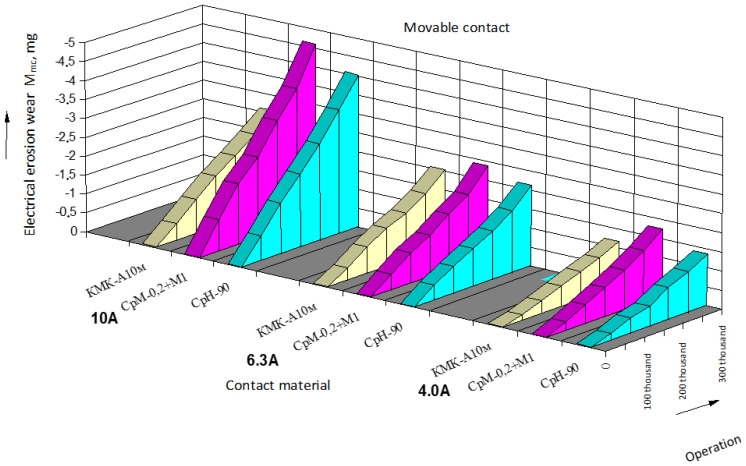
Dependence of electrical erosion wear of serial movable (bridging) contact parts of PML starters.

**Figure 6:**
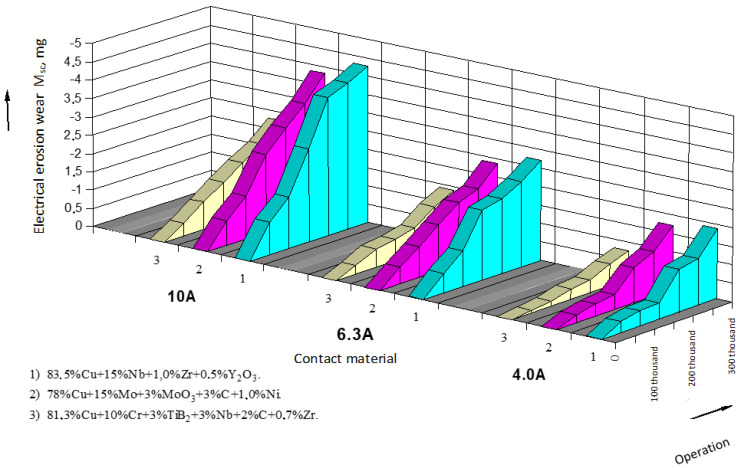
Dependence of electrical erosion wear of the experimental stationary contact parts of starters.

**Figure 7:**
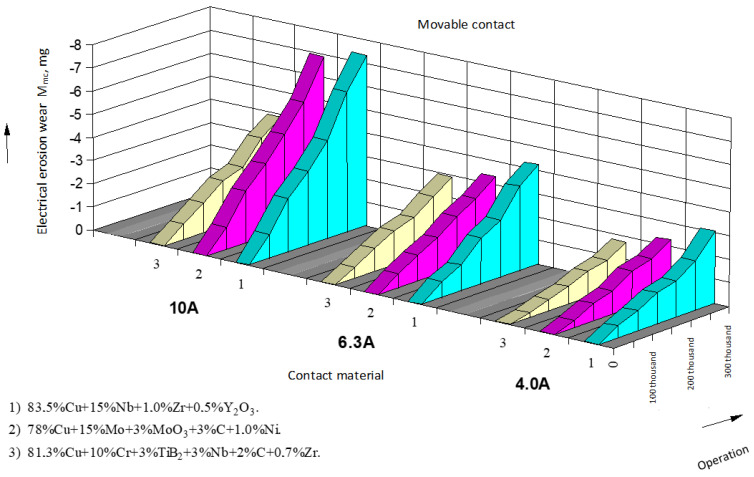
Dependence of electrical erosion wear of the experimental moving (bridge) contact parts of starters.

The results of data processing and wear intensity coefficients are presented in the form of tables, one of which is provided below (Table 1; for serial contacts, current—10 A). Based on the processed experimental data, the dependencies of electrical erosion wear for moving and stationary serial contact parts of starters based on silver (Figure 4 and Figure 5) as well as for the experimental contact parts based on copper (Figure 6 and Figure 7) are presented.

When switching alternating current (AC), the polarity of current flow through a contact continuously changes. Based on the experimental data, a negative (reduction in mass) wear intensity coefficient in both stationary and movable contact parts has been determined. However, movable contact parts experienced wear at a higher rate, approximately 10 to 27% more. This phenomenon is typical for AC contacts and can be explained by the fact that the temperature of movable bridges is higher than that of stationary contacts, by about 25 to 30 °C. The mass of movable bridge-type contacts decreased slightly more than the mass of stationary contacts because the processes during arc erosion are accompanied by more intense evaporation and material splattering, due to the higher temperature [17,20].

It has been established that with an increase in the commutation current, erosion wear also increases. This is due to the fact that with the increase in current, the factors related to a plasma arc play a more significant role in contact processes, leading to an increase in arc erosion wear through the growth of electrical arc energy. Additionally, arc erosion is significantly affected by the phase composition and the structure of contact material, as the arc channel is anchored to the structural components with low thermal and electrical conductivity [17,19].

Contacts KMK-A10m (starter PML 2100.V) exhibit greater erosion resistance. The resistance of KMK-A10m material is higher by 13–30% compared to contacts made from CpH-90 material and 35–45% higher than bimetallic contacts made from CpM-0,2 + M1 material. High electrical erosion resistance of KMK-A10m contacts in the research was achieved through the material structure and the peculiarities of cadmium oxide (CdO) material [20,22].

**Figure 8:**
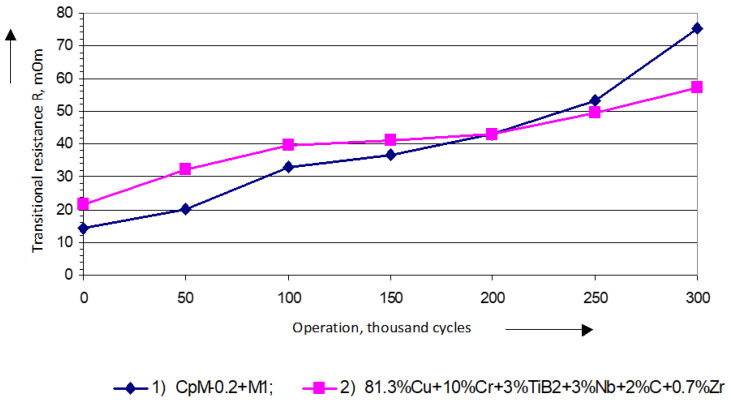
Dependence of transitional resistance of the contacts of PML-1100 O4 starters with serial (1) and experimental contacts (2) (test environment: a mixture of H_2_S (25 mg·m^−3^)+NH_3_ (30 mg·m^−3^).

As a result of processing the statistical data, the experimental dependencies of transient contact resistance on the number of switching cycles were obtained, which are presented in Figure 8. These dependencies were approximated by a first-degree polynomial, and the following expressions for the mathematical expectation of contact resistance were determined:

- For serial contacts CpM0,2 + M1:
  (3) $$R_{c}\left(n\right)=14.22+0.191\cdot n$$
- Root-mean-squared approximation error Ec = 2141;
- For experimental contacts 81.3% Cu + 10% Cr + 3% TiB_2_ + 3% Nb + 2% C + 0.7% Zr:
  (4) $$R_{д}\left(n\right)=21.84+0.128\cdot n$$
- Root-mean-squared approximation error Ec = 1855.

Research on determining the contact resistance of contacts when exposed to an environment with chemically active components revealed that the initial value of the transitional resistance of contacts increased with the duration and the number of commutation cycles. The resistance of serial CpM-0,2 + M1 contacts increased by almost five times compared to the initial resistance, while the resistance of the experimental contacts increased slower than that in silver-based materials, by about 2.7 times. This can be explained by the fact that the boundary layer consists of films that form on the contacts and have a homogeneous composition, and their thickness increases with the duration of their exposure to the environment. Such a film structure on a contact surface contributes to a reduction in transitional resistance by breaking down localized particles and establishing a reliable metallic contact. Copper oxide films are known to form even at room temperature, and their thickness does not exceed 5 µm. Such films protect the copper from further oxidation by oxygen and stabilizes contact resistance.

Arc erosion is a complex physical phenomenon influenced by various factors, including electrical, material, mechanical and environmental factors. It can be observed that the impact of electrical parameters and the number of commutation cycles on arc erosion is fairly well understood [2,17]. However, there is relatively limited knowledge about the impact of the presence of aggressive impurities in the surrounding environment (hydrogen sulfide, ammonia, etc.), where the arc occurs, on the extent of arc erosion.

**Figure 9:**
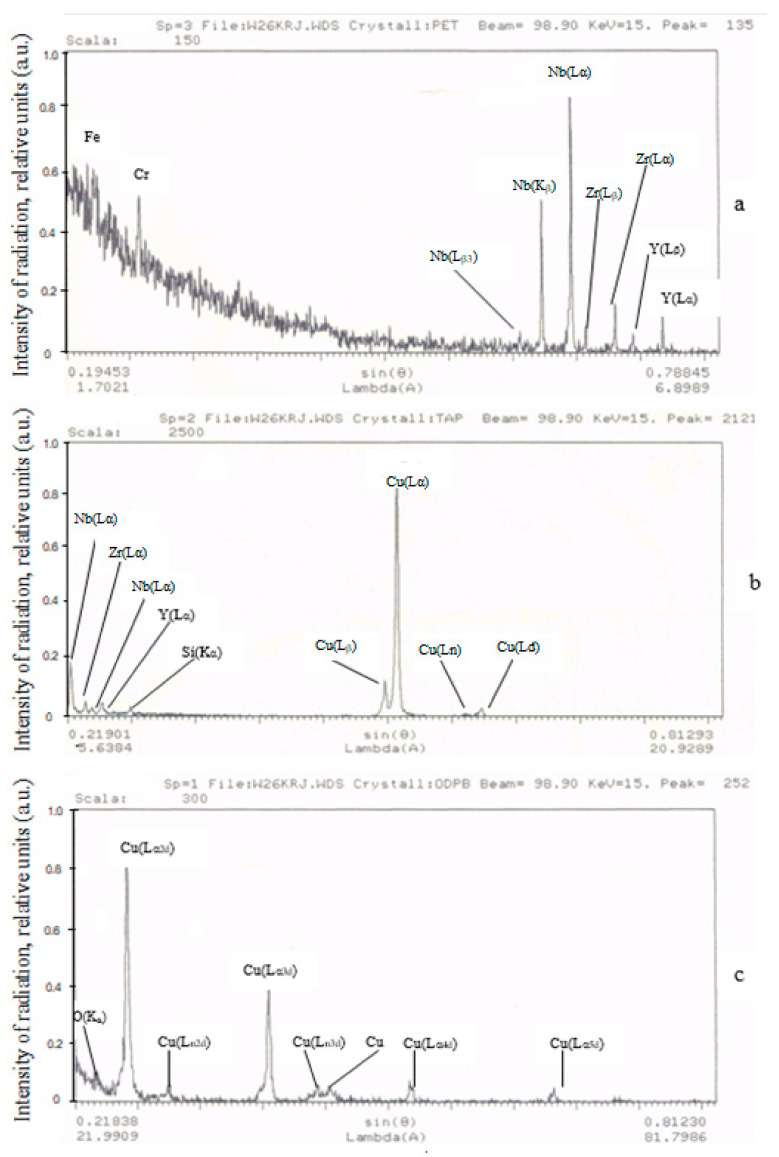
Spectra of radiation from the components of contact materials 83.5% Cu + 15% Nb + 1.0% Zr + 0.5% Y_2_O_3_ obtained using crystal analyzers: (**a**) RET; (**b**) TAR; (**c**) ODPB.

The conducted X-ray spectral analysis of the contacts allows for the qualitative chemical composition and element distribution in the surface layers of the working zone of the experimental contact material to be identified (see Figure 9). The measurement principle used here is the measurement of X-ray fluorescence induced by a focused electron beam. In Figure 9a, technological impurities, such as Fe and Cr elements, as well as the components of the contact material: niobium, zirconium and yttrium were identified. The presence of Fe and Cr elements can be attributed to the accidental deposition of these materials from the structural elements of a starter. The data in Figure 9b,c indicate that only the elements constituting contact material were present on the working surfaces, although those elements were identified with varying intensities of radiation and different phase compositions. Additionally, Figure 9c shows oxygen lines of low intensity.

**Figure 10:**
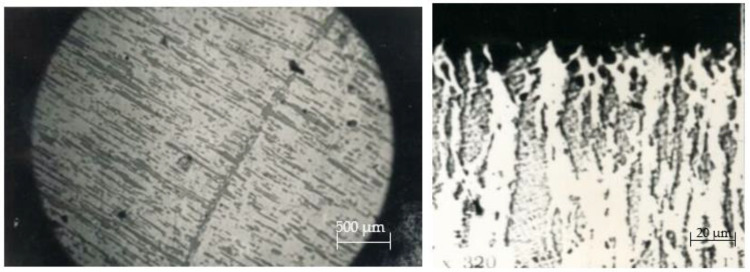
Microstructure of a stationary contact part of PML-1100 O_x_4A starter after 150,000 switching cycles. Category AC-3 (U = 380 V; I = 10 A). Material: CpH-90.

The microstructure of serial contact material CpH-90 essentially consists of a uniform fine-dispersed mixture of silver and nickel phases, the particles of which elongated parallel to the wire-drawing direction, providing an anisotropy of the physical characteristics (see Figure 10). The structure of pseudo-alloy CpH-90, especially the surface layers subjected to multiple thermal effects of the electric arc, changed. As one approached the working surface, the grain size increased. Due to the nickel content (10%) and the orientation of its individual particles, various types of structures were formed in the rupture zone. In this case, on the working surface, a structure with small Ni inclusions inside the low-melting component Ag was formed. Therefore, a relatively high resistance to burning combined with low contact resistance (which is highly suitable for low currents, of approximately 10 A). The depth of the layer in which microstructural changes occurred was in the range of 0.08 to 0.12 mm. In that process, nickel oxidized directly on the contact surface and up to a depth of 0.1 mm.

On the working surface, shallow pores and craters appeared in the place where silver and agglomerations of the refractory nickel grains were located. During electrical contact operation, there was melting and an intense evaporation of the low-melting component—silver—from the working surface of a movable contact part. This is because the evaporation temperature of silver (2210 °C) is significantly lower than the evaporation temperature of nickel (2730 °C) (see Figure 10 ×320). As a result, a dispersed rough surface is formed, and its degree of roughness depends on the current magnitude (see Figure 1 and Figure 3) and the number of switching cycles (see Figure 3a).

Large protrusions appeared on the erosive surface and light inclusions were located within structural depressions, which consisted of pure silver, according to X-ray spectral analysis. Chemical analysis of the surface layers showed a significant increase in the amount of nickel on the working surface (from 10% to 20–25%), indicating the primary nature of silver evaporation from the contact material surface during the electrical current switching process [18,19,20].

**Figure 11:**
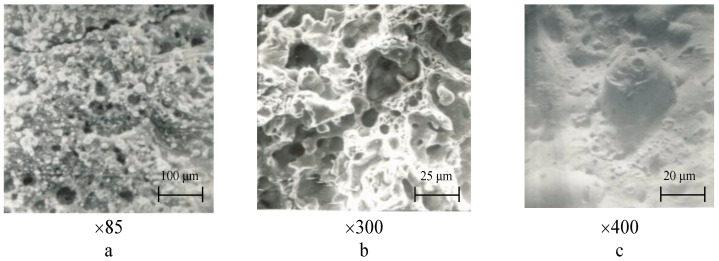
Morphology of the working surface of contact parts made from CpH-90 material after 150,000 switching cycles (**a**) magnification 85, (**b**) magnification 300, (**c**) magnification 400.

The conducted detailed research on the working surfaces of contact parts revealed that, due to the action of an electrical arc, both a low-melting component of the composition, which is silver, and nickel grains melted (Figure 11a). This was confirmed by the presence of characteristic solidification protrusions on the edges of nickel grains (Figure 11b). Elongated nickel grains had a cone shape, which was typical for bridging transfer (Figure 11c). On the tops of some nickel grains, the areas of viscous detachment formed, indicating material damage after contact adherence [14,19].

The obtained results allow us to state that contact at the last moment of switching is made at the points of a high-melting component, which determines the tendency of CpH-90 material to weld.

**Figure 12:**
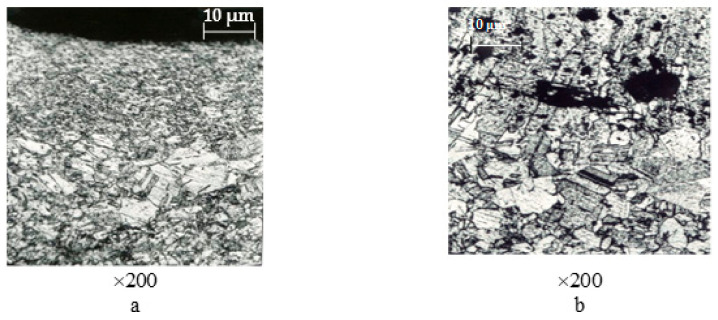
Microstructure of stationary (**a**) and moving (**b**) contact parts of PML-1100 0 × 4 starter after 150,000 switching cycles (category AC-3 (U = 380 V; I = 10 A); material CpM-0,2 + M1).

During current switching with contacts made from CpM-0,2 + M1 material, there was a noticeable burning and melting of the working surfaces of contact parts. Metallographic analysis showed that the microstructure of surface layers of stationary and movable contact parts (Figure 10) differed in its structure and underwent significant changes during operation. It was evident that during current switching, the material of a movable contact part was heated to its boiling temperature, partially transferred to the surface of a cooler stationary contact part (Figure 12a), and the rest re-deposited on the surface of a movable contact, creating voids (seen as black craters in Figure 12b) and columnar grains perpendicular to the working surface of a contact part.

The surface of a stationary contact part was covered with tiny grains of metal vapor from a movable contact part, which crystallized when an electrical arc was extinguished. Additionally, on the surface where those metal vapor deposits were settled, enlarged silver grains were observed. Those larger grains were formed as a result of recrystallization due to the elevated temperature.

When replacing serial contact material with the experimental one, a series of tests were conducted to determine technical parameters of the experimental starters and to objectively assess their technical performance. The conducted testing of the experimental contacts for commutation wear resistance showed that for composite contact materials, the linear law of electrical erosion wear was maintained, but the intensity coefficient of electrical erosion wear increased by 15–30% (Figure 6 and Figure 7). This can be explained by the fact that under the action of an arc, copper contacts oxidize more than in series-produced contacts, and thermal conductivity of the experimental materials is lower than that of silver-based materials, leading to a significant local overheating of copper. It could be observed that in the experimental contacts, the extent of burning was greater, and traces of material erosion were more prominent on the working surface [1,2,31].

**Figure 13:**
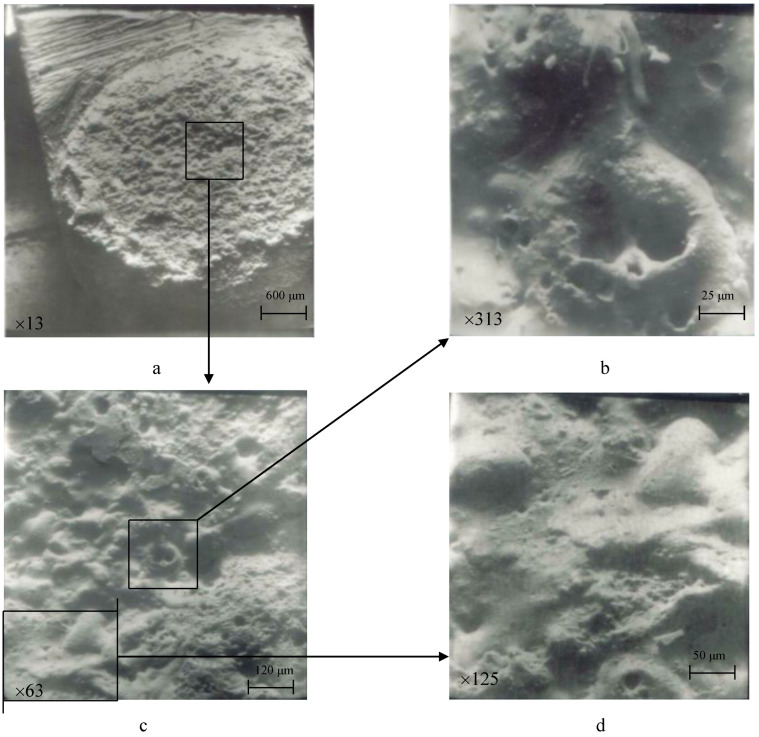
Surface morphology of stationary contact parts after 150,000 switching cycles (material 83.5% Cu + 15% Nb + 1.0% Zr + 0.5% Y_2_O_3_). (**a**) magnification 13, (**b**) magnification 313, (**c**) magnification 63, (**d**) magnification 125.

**Figure 14:**
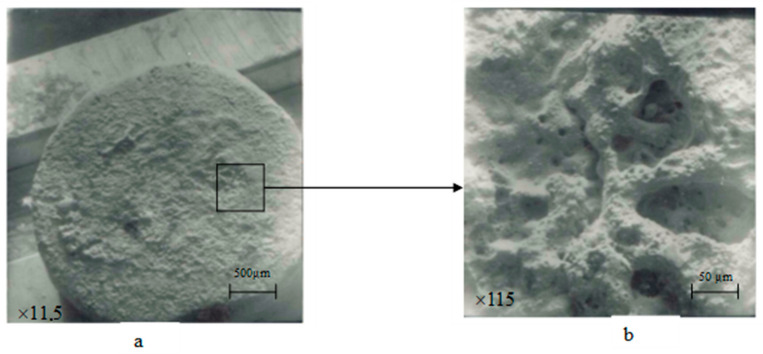
Surface morphology of movable contact parts (bridges) after 150,000 switching cycles (material 83.5% Cu + 15%Nb + 1.0%Zr + 0.5%Y_2_O_3_). (**a**) magnification 11.5, (**b**) magnification 115.

In the images of stationary contacts made from the experimental material, a high current density was observed, which led to the formation of arc streams, causing the erosion of the other electrode. Since the current was alternating, we had a similar pattern of erosion on both electrodes: molten material along with material transfer in the vapor phase (Figure 13b and Figure 14).

**Figure 15:**
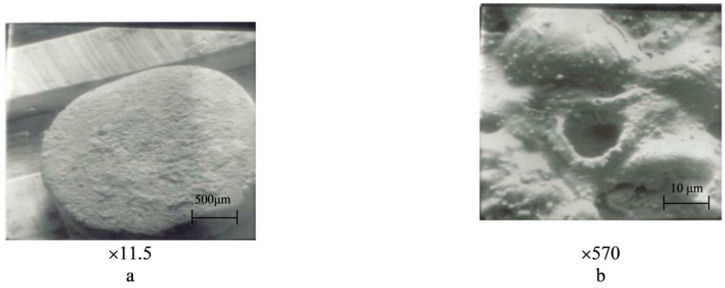
Surface morphology of movable contact parts after 150,000 switching cycles (material: 81.3% Cu + 10% Cr + 2.0% TiB_2_ + 3.0% Nb + 2.0% C + 0.7% Zr). (**a**) magnification 11.5, (**b**) magnification 570.

**Figure 16:**
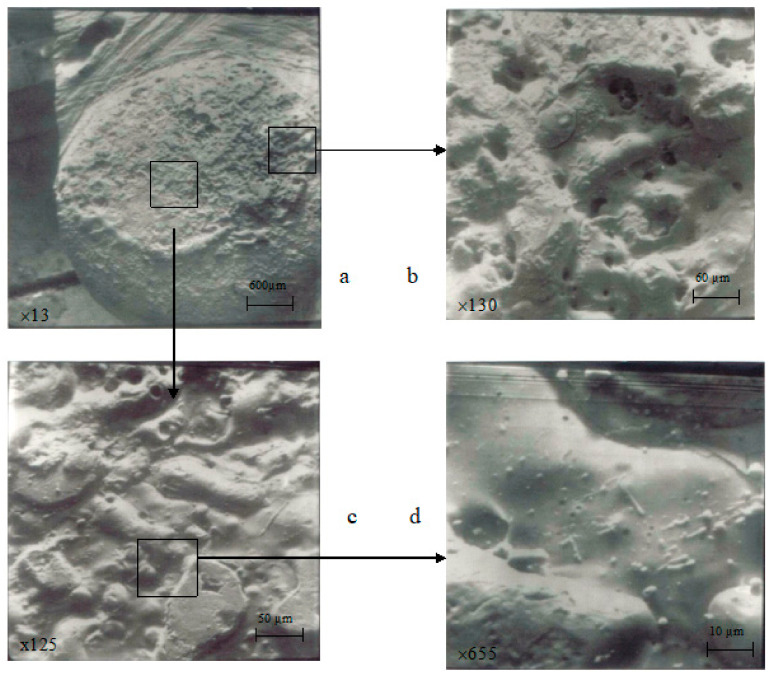
Surface morphology of stationary contact parts after 150,000 switching cycles (material: 81.3% Cu + 10% Cr + 2.0% TiB_2_ + 3.0% Nb + 2.0% C + 0.7% Zr). (**a**) magnification 13, (**b**) magnification 130, (**c**) magnification 125, (**d**) magnification 655.

Cathode-type spots were predominantly observed, mainly in the form of small dots (illustrated in Figure 14b, Figure 15b and Figure 16b,d). In some cases, there were instances of a combination of cathode- and anode-type spots (as seen in Figure 14b, a large oval with small dot-like inclusions).

The destruction of electrodes occurred both under the action of anode and cathode spots as well as under the influence of plasma jets of the electric arc (single torches—Figure 13c, Figure 14b and Figure 16b; a large-sized crater—Figure 16a,b).

The contact material melted, and the liquid phase transferred (forming a rippled surface and bumps) while cathode plasma torches appeared (mainly on stationary contacts—Figure 13b), leading to significant damage on the movable contacts (bridge contacts—Figure 14). On the movable contacts, isolated spots could be observed in the places where plasma jets exited. Considering that the switching occurred with alternating current, both movable and stationary contacts experienced material losses and transfer. Cathode torches from the stationary contacts damaged the movable contacts more actively. Therefore, it is recommended to use a more erosion-resistant material for movable contacts [2,8].

The increase in Nb content to 5% or more led to the enhanced evaporation of copper due to significant differences in the thermophysical properties of components during the electric arc discharge. As a result, the working surface became enriched with niobium, and copper was found in small quantities in the form of small spherical particles, primarily deposited on the peripheral areas (Figure 13b, Figure 15b and Figure 16d).

Increasing the chromium content in Cu–Cr composition had a more favorable impact on the erosion resistance of contacts compared to Nb due to a smaller difference in their thermophysical properties, despite the presence of numerous areas affected by the electric arc (Figure 13 and Figure 16). Throughout the contact surface, there was copper present as a main component, generally in the form of a solidified melted metal upon cooling (Figure 16c,d). The majority of molten copper formed under the arc supporting the spots was splattered onto the edges of a contact part, where it solidified into large clusters on the tangential and extended beyond the contact limits, including its escape in the vapor phase. As a result, the working surface of a contact part can be characterized by numerous areas influenced by the electric arc.

Such a pattern may possibly be explained by well-known data regarding arc burning conditions: the minimum arc burning voltage (45 V) is observed on graphite contacts compared to the major metals used in contact technology [22,31].

**Figure 17:**
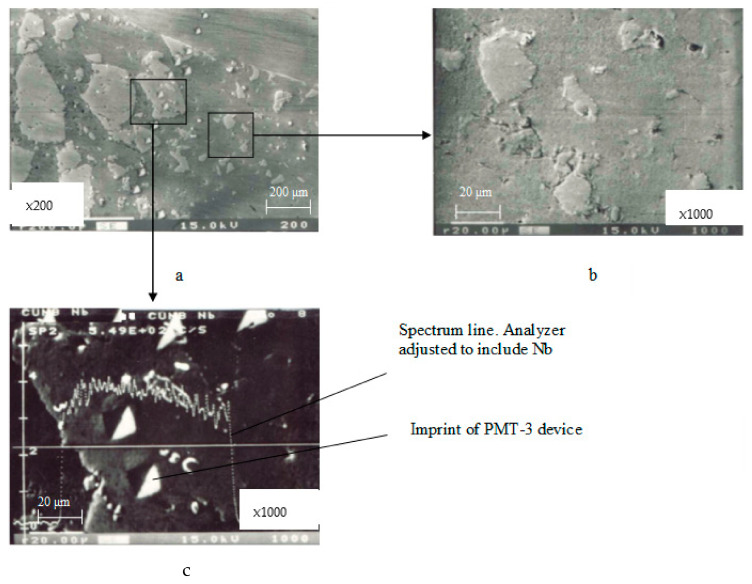
Microstructure of the working surface of contacts. Material: 83.5% Cu + 15% Nb + 1.0% Zr + 0.5% Y_2_O_3_; (**a**) distribution of microhardness across the contact cross-section; (**b**) inclusions of Zr and Y_2_O_3_ particles; (**c**) inclusions of Nb particles.

The copper matrix was reinforced with small-sized inclusions of yttrium oxide (Y_2_O_3_) and zirconium (Zr). The hardness of the matrix was 85–90 kg·mm^−2^ and increased to 100–115 kg·mm^−2^ on Y_2_O_3_ and Zr inclusions. The presence of niobium (Nb) inclusions further increased the hardness to 165–190 kg·mm^−2^, with a smaller imprint size as indicated with the PMT-3 device (Figure 17a,c).

## 4. Conclusions

1. The results of the experimental commutation tests on the contacts of electromagnetic starters allowed us to establish the decisive influence of a current’s magnitude on the electrical wear of contacts. The magnitude of electrical erosion wear is the smallest in CpH-90 material; CpM-0,2 + M1 material is less erosion resistant and its erosion wear is 1.2–1.3 times greater than that of the contacts made of the CpH-90 material.
2. In the experimental contacts based on copper (Cu + Cr + TiB_2_ + Nb + C + Zr), the magnitude of electrical erosion wear increased by 15–30% compared to the series-produced contacts due to a significant local overheating of copper in the experimental contacts. In this case, the wear law remains linear.
3. The erosion wear of starter contacts occurs due to the action of plasma streams from alternating current electrical arcs, which was morphologically confirmed via the photographs of the working surface of the contacts. Cathode torches from the stationary contact damaged the moving contact more actively. Thus, it is advisable to recommend a more erosion-resistant material for a moving contact.
4. A significant volume of graphite in standard Cu–C compositions prevents the welding of contact surfaces under the influence of an electric arc discharge, both due to the cooling effect associated with the formation of large volumes of volatile oxides (CO, CO_2_), and the film deposited from the vapor phase on the working surfaces of contacts. This disrupts the already weak adhesion strength of copper framework sectors, leading to a substantial localized overheating of copper and, consequently, a significant damage to a contact part (increased wear).
5. In order to create contact materials with the necessary working properties for electromagnetic starters used in agriculture, it is advisable to select composite materials based on copper, consisting of such components as Cr, Nb, Zr, Y_2_O_3_, C, TiB_2_, etc. [16,17].

## Figures and Tables

**Table 1:** The results of wear intensity of contact components of starters as a function of the number of commutation cycles (*I_nom_* = 10 A).

Contact Material	Number of Cycles, Thousands	Contact Component Number and Mass Change, mg											Wear Per One Cycle k, 10^−6^ g/Cycle
		Stationary Contact Components							Movable Bridges				
		1	2	3	4	5	6	Average Wear	1–2	3–4	5–6	Average Wear	
CpH-90	50	−0.45	−0.50	−0.55	−0.45	−0.50	−0.55	−0.50	−0.65	−0.65	−0.65	−0.65	−10.41 stationary
	100	−1.00	−1.05	−1.00	−1.10	−0.95	−0.95	−1.01	−1.25	−1.50	−1.25	−1.33	
	150	−1.50	−1.55	−1.50	−1.65	−1.50	−1.50	−1.53	−1.80	−2.15	−1.80	−1.92	
	200	−1.90	−1.95	−2.10	−2.15	−2.10	−2.15	−2.06	−2.35	−2.70	−2.60	−2.55	
	250	−2.55	−2.50	−2.55	−2.60	−2.60	−2.75	−2.59	−3.05	−3.25	−3.50	−3.27	−13.12 moving
	300	−3.10	−3.15	−3.20	−3.15	−3.25	−3.20	−3.18	−3.85	−4.10	−4.15	−4.03	
	absolute wear							−3.18				−4.03	
CpM-0,2 + M1	50	−0.70	−0.75	−0.60	−0.75	−0.60	−0.65	−0.68	−0.85	−0.8	−0.8	−0.82	−12.05 stationary
	100	−1.30	−1.40	−1.05	−1.45	−1.20	−1.25	−1.06	−1.65	−1.65	−1.50	−1.60	
	150	−2.00	−2.00	−1.70	−2.05	−1.80	−2.00	−1.93	−2.10	−2.10	−2.15	−2.12	
	200	−2.55	−2.55	−2.30	−2.55	−2.30	−2.65	−2.48	−3.00	−2.90	−2.85	−2.92	
	250	−3.05	−3.15	−3.10	−3.15	−2.90	−3.15	−3.08	−3.80	−3.60	−3.50	−3.60	−14.90 moving
	300	−3.65	−3.70	−3.95	−3.85	−3.65	−3.95	−3.79	−4.55	−4.65	−4.60	−4.60	
	absolute wear							−3.79				−4.60	
KMK-A10m	50	−0.40	−0.50	−0.40	−0.35	−0.35	−0.30	−0.38	−0.4	−0.5	−0.45	−0.45	−7.70 stationary
	100	−0.75	−0.95	−0.80	−0.70	−0.85	−0.65	−0.78	−0.85	−0.9	−1.0	−0.92	
	150	−1.10	−1.30	−1.25	−1.10	−1.20	−0.95	−1.15	−1.2	−1.45	−1.45	−1.37	
	200	−1.40	−1.60	−1.70	−1.45	−1.50	−1.30	−1.49	−1.6	−1.85	−1.8	−1.75	
	250	−1.90	−1.95	−2.05	−1.85	−1.90	−1.85	−1.92	−2.1	−2.2	−2.1	−2.13	−8.69 moving
	300	−2.35	−2.45	−2.40	−2.25	−2.40	−2.25	−2.35	−2.65	−2.6	−2.8	−2.58	
	absolute wear							−2.35				−2.58	

## Data Availability

All data generated or analyzed during this study are included in this published article.
